# The Psychiatric Consequences of Long-COVID: A Scoping Review

**DOI:** 10.3390/jpm12111767

**Published:** 2022-10-26

**Authors:** Gaia Sampogna, Matteo Di Vincenzo, Vincenzo Giallonardo, Francesco Perris, Antonio Volpicelli, Valeria Del Vecchio, Mario Luciano, Andrea Fiorillo

**Affiliations:** Department of Psychiatry, University of Campania “L. Vanvitelli”, 80138 Naples, Italy

**Keywords:** long-COVID syndrome, cognitive impairment, depression, fatigue, anxiety

## Abstract

The COVID-19 pandemic has represented a new form of traumatic event, affecting the general population worldwide and causing severe disruption of daily routine. A new urgent concern is related to the burden associated with COVID-19 symptoms that persist beyond the onset of infection, the so-called long-COVID syndrome. The present paper aims to: (1) describe the most frequent psychiatric symptoms reported by patients affected by long-COVID syndrome; (2) evaluate methodological discrepancies among the available studies; (3) inform clinicians and policy-makers on the possible strategies to be promoted in order to manage the psychiatric consequences of long-COVID syndrome. Twenty-one papers have been included in the present review, mostly with a cross-sectional or cohort design. Significant heterogeneity of long-COVID syndrome definitions was found. The presence of psychiatric symptoms was evaluated with very different assessment tools. The most common psychiatric symptoms of the long-COVID syndrome included fatigue, cognitive disturbances/impairment, depression, and anxiety symptoms. The rate of fatigue varied from 93.2–82.3% to 11.5%, cognitive impairment/cognitive dysfunction from 61.4% to 23.5% and depressive-anxiety symptoms from 23.5%to 9.5%.

## 1. Introduction

The COVID-19 pandemic has represented a new form of traumatic event [1,2,3,4,5,6], being a completely unexpected event, affecting the whole population worldwide and causing severe disruption of daily life [7,8,9,10,11,12,13,14]. The pandemic and its related containment measures have had a serious negative impact on the mental health of the general population [15,16,17,18,19,20,21,22] and of special target groups [23,24,25,26,27,28,29,30,31,32]. The development and dissemination of vaccination campaigns have significantly reduced the mortality rates due to the virus worldwide [33,34,35], although the World Health Organization has not yet declared the end of the pandemic crisis [36,37].

A new urgent concern is related to the burden associated with COVID-19 symptoms persisting beyond the onset of the infection, called COVID-19 long haul symptoms or post-COVID-19 syndrome. This condition includes a wide range of new and returning health problems that people experience after the infection. The post-COVID-19 syndrome can be identified and diagnosed at least four weeks after the infection and can develop in anyone who has been infected [38,39,40,41].

The National Institute for Health and Care Excellence (NICE) guidelines define the post-COVID-19 syndrome as “signs and symptoms that develop during or after an infection consistent with COVID-19, continue for more than 12 weeks (3 months) and are not explained by an alternative diagnosis” [42]. However, the term “long COVID” is used to refer to the protracted illness, lasting from 4 [43] to 12 weeks [44] after the acute illness and during recovery. In fact, no universal consensus has been reached so far on the definition of this clinical condition, and other terms are used, such as synonyms, including “post-acute COVID-19”, “ongoing symptomatic COVID-19”, “chronic COVID-19”, “post COVID-19 syndrome” and “long-haul COVID-19”.

The long COVID syndrome can be due to several aetiopathogenetic factors, including the brain localization of the virus, the presence of stroke, hypoxia, hyperinflammation, the persistent presence of SARS-CoV-2, or hypoxia-induced mitochondrial dysfunction [45,46]. The COVID-19 disease is characterized as a cytokine release syndrome, with elevated serum concentrations of interleukin-6 and other inflammatory cytokines, which correlate in a dose–response manner with respiratory failure, adverse respiratory distress syndrome, and other clinical outcomes. It is likely that an immuno-inflammatory dysregulation significantly contributes to acute and post-acute psychiatric and cognitive symptoms in COVID-19 patients [47].

However, there are no laboratory tests to diagnose the post-COVID-19 condition, and the wide variety of symptoms ranging from respiratory difficulties to neuropsychiatric symptoms could derive from other health problems, making it difficult for healthcare professionals to recognize and appropriately manage the syndrome. Although several reviews and meta-analyses have already been published [48,49,50], the clinical picture of the post-COVID condition is still not clear.

This scoping review aims to: (1) describe the most frequent psychiatric symptoms presented by patients with the long-COVID syndrome; (2) evaluate methodological discrepancies among the available studies; (3) inform clinicians and policymakers on possible strategies in order to efficiently manage the psychiatric consequences of long-COVID syndrome.

## 2. Materials and Methods

This review was performed in five stages: the definition of the problem, the literature search, data evaluation, data analysis, and the presentation of findings.

**Figure 1:**
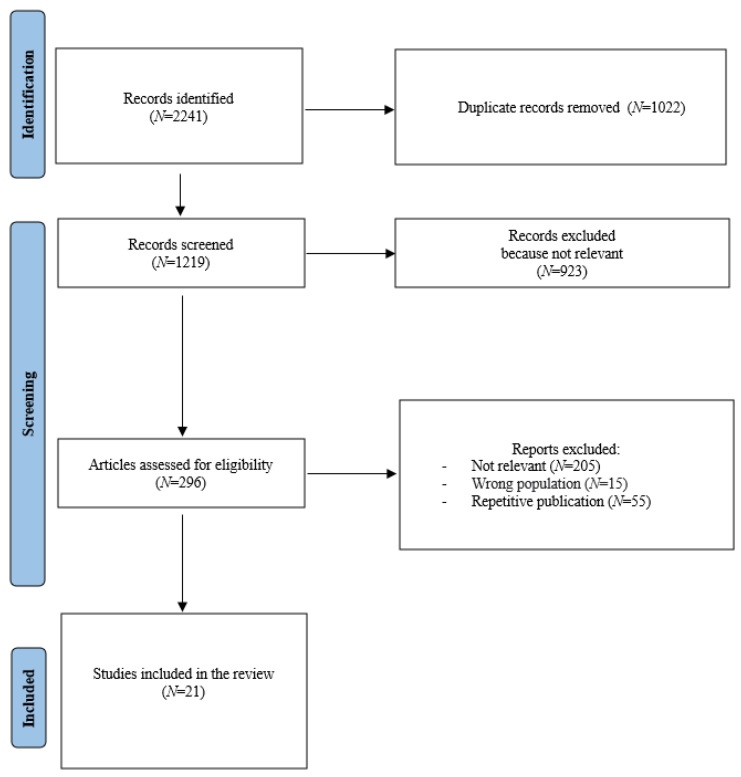
PRISMA flow diagram of selection of studies for inclusion in the review.

The search terms “long-term symptoms”, “long-COVID”, “psychiatry”, “mental disorders”, “post-COVID condition”, “depression”, and “anxiety”, were entered into ERIC, MEDLINE, PsycARTICLES, PsycINFO, SCOPUS, and PUBMED (Figure 1). Terms and databases were combined using the Boolean search technique, which consists of a logical information retrieval system (two or more terms combined to make searches more restrictive or detailed).

In this scoping review, we have considered published case reports, observational, case–controls, cohorts, randomized control trials (RCT), as well as retrospective and prospective real-world experience studies of COVID-19 infection. Publications were identified by searching electronic databases and the reference lists of selected articles. The search was limited to studies published in English. The electronic database search was conducted starting from the publication of the systematic review and meta-analysis of Badenoch et al. [51], in December 2021. Only studies focused on adult populations (aged 18 or more) have been included. Studies on underaged children and/or adolescents were excluded since the available prevalence data of long COVID syndrome in such a population suffers from extreme heterogeneity [52,53], requiring a different management plan compared to the adult population [54]. Reviews were excluded from the analysis, but their reference lists were searched in order to identify relevant primary publications.

### Study Selection and Data Extraction

Authors screened the articles identified by the searches and then performed a full-text review of those that appeared relevant to the research topic based on titles and abstracts. Only studies dealing with neuropsychiatric/psychiatric symptoms in patients infected by COVID-19 were included. The studies were then assessed independently by two reviewers (GS and MDV) to extract the main data. The kappa measure of agreement was 0.81, confirming an almost complete agreement.

Disagreements that arose between the reviewers were solved through discussion, and in the case of continued disagreement, with the assistance of a third senior researcher (AF). Data on study characteristics (author, year, country), study design and inclusion criteria, the definition of the post-COVID syndrome, assessment tools, and main findings were extracted.

## 3. Results

A total of 2241 studies were identified; of these, 1022 were duplicates and were thus excluded. Following the abstract screening, 296 full-text papers were evaluated, and 21 papers were included in the systematic review (Figure 1). Most studies had a cross-sectional or cohort design. Other studies were case–control (N = 3 studies), retrospective (N = 3) [55,56,57], case series (N = 1) [58], and case reports (N = 1) [59]. The majority of the studies were carried out in Europe (N = 16) (Table 1). The sample sizes of the studies varied from 30 [56] to 18,811 patients [57]. One study included only adult patients with subjective cognitive complaints following COVID-19 infection [60] (Table 2).

Almost all the studies included in the criterion contained laboratory-confirmed SARS-CoV-2 infections, as evidenced by a positive real-time reverse transcriptase polymerase chain reaction (PCR) among the selection criteria (Table 2). Alradini et al. [55] and Matsumoto et al. [70] collected data mainly by phone or on an online platform, and the presence of infection was self-declared by participants.

As regards the definition of “long-COVID syndrome”, we found significant heterogeneity among the studies. Ten studies lacked a clear, operational, and rigorous definition; in particular, Cacciatore et al. [61], Calabria et al. [60], Damanti et al. [64], De Las Penas et al. [65,66], Farooqui et al. [56], Stallmach et al. [72], and Voruz et al. [74] reported that recruited patients included those who had survived COVID-19 or who were discharged from a COVID-19 unit but did not provide a specific time frame for the evaluation of the presence of COVID-related symptoms. Additionally, Jozuka et al. [59], in their case report on the long-term consequences of COVID-19 infection, did not provide any temporal information.

The presence of psychiatric symptoms was evaluated with very different assessment tools; in particular, depression and anxiety symptoms were assessed with the Hospital Anxiety and Depression Scale (HADS) [61], the Patient Health Questionnaire (PHQ) [73], and the Generalized Anxiety Disorder-7 (GAD-7) [62]; cognitive impairment with the MoCA [56,61,62,68,72]; fatigue was evaluated with the Modified Fatigue Impact Scale [60] and Fatigue Severity Scale (FSS) [53]. Moreover, six studies out 21 (28.6%) [55,57,58,59,63,70] used ad hoc assessment tools or clinical interviews.

The most common psychiatric symptoms of the long-COVID syndrome included fatigue [55,58,59,62,66,68,69,72,73], cognitive disturbances/impairment [58,61,63,71,74], depression and anxiety symptoms [57,62,65,67,70,72,75]. The rate of fatigue varied from 93.2–82.3% [60,76] to 11.5% [55], cognitive impairment/cognitive dysfunction from 61.4% [61] to 23.5% [72], and depressive-anxiety symptoms from 23.5% [60] to 9.5% [67].

## 4. Discussion

This scoping review aims to provide an updated estimation of the most frequent psychiatric symptoms and manifestations in patients with the long-COVID syndrome.

Although precise estimations about the absolute risk are still difficult to provide, our findings confirm that the most prevalent psychiatric symptoms in the long-COVID syndrome include fatigue, cognitive impairment, and depression and anxiety symptoms [76,77].

Cognitive impairment, including difficulties with concentration, memory, receptive language, and/or executive functions, has been reported in several people who have had a symptomatic COVID-19 infection. Psychiatric symptoms and cognitive impairment can develop and persist months after the infection, and their development may partly be the result of somatic, functional, or psychosocial consequences of the disease. In particular, coronaviruses can induce cognitive, emotional, neurovegetative, and behavioral dysregulation due to direct neurological injuries through hypoxic damage and neuroinvasion [50]. In addition to this, the systemic immune activation seen in COVID-19 can significantly contribute to the mental health toll even months after the initial disease. Coronaviruses can also induce cognitive, emotional, neurovegetative, and behavioral dysregulation through a direct neurological injury characterized by hypoxic damage and neuroinvasion. Moreover, neuroinflammation might play a crucial role in the development of depressive and cognitive symptoms, as confirmed in longitudinal studies carried out with patients with high levels of inflammatory markers associated with long-term cognitive decline, including the deterioration of memory and executive functions [49,50].

However, the long-term symptoms reported by COVID-19 survivors are likely to be similar to those observed in survivors of SARS, where at least 30% of them reported a significant reduction in mental health one year later [78].

Memory impairment represents a common feature of the long-COVID syndrome, and the effect of SARS-CoV-2 on cognition may be related to the vulnerability of various CNS cells to the virus and its direct infiltration of the CNS. The viral attachment of host cells results from the binding of the S1 subunit of the S protein, one of four structural proteins of the SARS-CoV-2 virion, to the angiotensin-converting enzyme 2 (ACE2) receptor on cell surfaces, with a subsequent intracellular entry of the viral genome occurring after the fusion of the viral and host cell membranes [79]. The neurotropism of SARS-CoV-2 should be mediated by the retrograde axonal transport following the invasion of peripheral olfactory neurons and/or by the breach of the blood–brain barrier following infection.

Cognitive impairment represents only one of the possible clinical manifestations of neuro-COVID, while other forms include meningoencephalitis, acute disseminated encephalomyelitis, encephalopathies with behavioral disturbances, seizures, and cerebrovascular disease.

Although data are still limited and preliminary, one of the main pathways behind cognitive impairment might be represented by the invasion of SARS-CoV-2 in the peripheral olfactory neurons, but this clearly requires further investigation and confirmation.

The rate of fatigue, which varies from 93.2% to 11.5%, lasts months after the respiratory symptoms are resolved, suggesting that CNS symptoms persist long after the acute infection [80].

Another aspect to be investigated is the association between the COVID-19 infection and the risk of dementia [81]. In fact, symptoms that commonly present in COVID-19, such as anosmia, have been previously associated with the onset of dementia and neurodegeneration [82].

The second aim of the present scoping review is to evaluate methodological discrepancies among the available studies. In particular, we found a high rate of methodological heterogeneity in included studies, with the majority of the studies adopting different assessment instruments for the evaluation of symptoms (e.g., for anxiety symptoms, Hospital Anxiety and Depression Scale (HADS) [70], the Patient Health Questionnaire (PHQ) [73], and the Generalized Anxiety Disorder-7 (GAD-7) [62]), or for the definition of the long-COVID syndrome (i.e., [56,60,61,65,66,72,74]).

Furthermore, the definition of the long-COVID syndrome is quite heterogeneous among the different studies. However, the lack of a consensus on the long-COVID syndrome itself represents a significant obstacle to the conduction of rigorous and reliable experimental studies in this field.

Finally, the last aim of the present review is to inform clinicians and policymakers on possible strategies in order to efficiently manage the psychiatric consequences of long-COVID syndrome. It must be acknowledged that the high rate of methodological heterogeneity among the included studies limits the development of appropriate interventions for the management of long-COVID symptoms. Therefore, it appears mandatory for policymakers, researchers, and clinicians to find an appropriate clinical definition, with consistent symptoms and diagnostic criteria in order to produce sound results. Further studies—both in vivo and in vitro—are needed to clarify the mechanisms and prevalence of long-COVID syndrome.

However, on the basis of the available data, the long-term psychological or adverse mental health consequences of COVID-19 have been widely recognized [83,84,85,86,87,88,89]. If neurodegeneration and new neuropsychiatric disorders happen in long COVID, this can become a major public health burden [90], even higher than that associated with acute illness. In order to reduce the long-term detrimental consequences of long-COVID syndrome, there is a need for effective treatments. As early as May 2020, The Stanford Hall consensus statement for post-COVID-19 rehabilitation [91] released recommendations for psychological and neurological sequalae. In particular, cognitive behavioral therapy (CBT) and Internet-CBT have been shown to be cost-effective for many psychiatric conditions while adhering to public health guidelines [90,91,92,93]. Other useful approaches to be tested may include psychoeducational interventions or stress-management techniques in order to support people in managing depressive/anxiety symptoms.

The present study has some limitations, which should be acknowledged. In particular, only studies written in the English language were included, which could have led to the exclusion of some national case reports. Moreover, the selection of studies focusing only on the adult population can be useful for informing ordinary clinical practice where the separation between young and adult psychiatric care is marked. However, this approach has prevented the identification of similarities in the long-COVID syndrome across different phases of lifespan.

## 5. Conclusions

Our scoping review clearly shows that the most common psychiatric symptoms of the long-COVID syndrome included fatigue, cognitive disturbances/impairment, depression, and anxiety symptoms. The rate of fatigue varied from 93.2–82.3% to 11.5%, cognitive impairment/cognitive dysfunction from 61.4% to 23.5% and depressive-anxiety symptoms from 23.5% to 9.5%. Moreover, several methodological discrepancies among the available studies have been identified in terms of the type of assessment tools adopted, the definition of the long-COVID syndrome, and the type of inclusion criteria. The physiopathological mechanisms of brain invasion are still far from being elucidated, but new studies are coming with an in vivo exploration through fMRI and PET techniques. Therefore, it appears mandatory for policymakers, researchers, and clinicians to find an appropriate clinical definition, with consistent symptoms and diagnostic criteria in order to produce sound results. Further studies—both in vivo and in vitro—are needed to clarify the mechanisms and prevalence of long-COVID syndrome.

## Figures and Tables

**Table 1:** Country representation of the included studies (N = 21).

Spain	5
Saudi Arabia	3
Japan	3 *
Italy	3
USA	3
Switzerland	2 *
Egypt	1
France	1
Germany	1

* One study has been conducted in Switzerland and Japan.

**Table 2:** Main characteristics of the included studies (N = 21).

Author(s), Year, Country, Study Design	Sample Size	Inclusion Criteria	Definition of Post-COVID Syndrome	Assessment Tools	Main Results
Alghamdi et al. (2022), Saudi Arabia [38] Online survey	N = 2218 participants	Laboratory-confirmed SARS-CoV-2 infection, as evidenced by a positive real-time reverse transcriptase-polymerase chain reaction test result.	Persistent neuropsychiatric disorders and conditions affecting the peripheral nerves from <1 to >6 months after COVID-19 infection.	Ad hoc questionnaire	Post-COVID neuropsychiatric symptoms, including altered cognitive skills, anosmia and dysgeusia, tinnitus, depression, and sleep disorders, were recorded in 18.9–63.9% of the participants with COVID-19.
Alradini et al. (2022), Saudi Arabia [55] Multicenter, retrospective cross-sectional study	N = 1000 participants	All patients with confirmed SARS-CoV-2 infection diagnosed At least 18 years old	Signs and symptoms that developed during or after an infection consistent with COVID-19, continuing for more than 12 weeks, and not explained by an alternative diagnosis.	Clinical records/telephone interview	Most common-to-late symptoms include loss of smell, loss of taste, fatigue, shortness of breath, and cough (52.4%, 31.1%, 11.5%, 10.2%, and 8.9% of patients with late symptoms, respectively).
Cacciatore et al. (2022), Italy [61] Cohort study	N = 105 patients	Patients who survived COVID-19 and were discharged from the COVID-19 Unit of the ASST Spedali Civili Hospital	No clear definition	WHODAS-12 Hospital Anxiety and Depression Scale (HADS) Pittsburgh Sleep Quality Index (PSQI) Montreal Cognitive Assessment (MoCA) Cumulative Illness Rating Scale (CIRS) COVID-19 severity	The most common symptoms at follow-up were sleep disturbances, cognitive dysfunctions, and fatigue. Cognitive dysfunction, anxiety, fatigue, and hyposmia/hypogeusia explained 28.8% of WHODAS-12 variation.
Calabria et al. (2022), Spain [60] Cross-sectional study	N = 136 patients with subjective cognitive complaints after SARS-CoV-2 infection	Having had COVID-19 symptoms and confirmed positive for SARS-CoV-2 via polymerase chain reaction (PCR) and/or serology (anti-SARS-CoV2 IgM or IgG) Being referred for neuropsychological assessment after reporting subjective cognitive complaints Being 18 + years old	No specific definition	Modified Fatigue Impact Scale Anxiety and Depression Scale (HADS) Frontal Systems Behavior Scale (FrSBe) European Quality of Life-5 Dimensions (EQ-5D) Brunnsviken Brief Quality of life scale (BBQ) World Health Organization Quality of Life—BREF (WHOQOL-BREF)	A total of 82.3% of individuals reported clinically significant levels of fatigue. Patients with clinically significant fatigue showed worse quality of life and poor daily functioning. Depressive symptoms and anxiety were reported in 23.5% and 35.3% of patients, respectively.
Chen et al. (2022), USA [62] Case-control study	Conga cohort: N = 200 COVID patients Control cohort; N = 342 patients.	Conga cohort: Patients testing positive for COVID-19 infection by respiratory swab or saliva sample RT-PCR with a minimum of four weeks from the date of confirmed COVID-19 infection or four weeks after the patient was discharged from the hospital Older than 18 years old Control cohort: patients enrolled in either the Georgia Cardiovascular Twin Study or the Georgia Stress and Heart study	Patients who were on a minimum of four weeks from the date of confirmed COVID-19 infection or four weeks after the patient was discharged from the hospital	Montreal Cognitive Assessment (MoCA) NIH Toolbox (NIH-TB) for the Assessment of Neurological and Behavioral Function studies. A University of Pennsylvania Smell Identification Test (UPSIT) A Waterless Empirical Taste Test (WETT) Patient Health Questionnaire-9 (PHQ-9) Generalized Anxiety Disorder-7 (GAD-7)	The most commonly reported COVID-19 symptom was fatigue (68.5%). In 25% of cases PHQ-9 criteria for depression were met. In 18% of cases GAD-7 criteria for anxiety were met. A total of 47% of patients met the criteria for mild cognitive impairment at MoCA.
Colizzi et al. (2022), Italy [63] Prospective study	N = 479 adult patients.	Consecutive patients, aged 18 years or older, admitted or seen on an outpatient basis at the hospital Infectious Disease Department, with a confirmed diagnosis of COVID-19.	“Post-COVID” symptoms had to be developed during or after COVID-19, and not to be explained by an alternative diagnosis in a follow-up period of 12 months after COVID-19 onset	Ad hoc questionnaire for evaluating clinical conditions	Significant increase was observed only for symptoms of psychiatric disorders (10.2%) and lack of concentration and focus (20%)
Damanti et al. (2022), Italy [64] Cross-sectional study	Three hundred and eighty-two patients	Patients aged 65 years or older, who attended a dedicated post-COVID-19 outpatient clinic. These patients were previously hospitalized for SARS-CoV-2 pneumonia in the Internal Medicine Department of the San Raffaele University Hospital, Milan, Italy and were discharged alive	Lack of specific definition	Medical examination, anthropometric measurements, strength assistance with walking, rising from a chair, climbing stairs, and falls (SARC-F) Short Physical Performance Battery (SPPB) test Mini Nutritional Assessment Short Form (MNA-SF) questionnaire EuroQol Group Health Questionnaire 5D-3L Visual Analog Scale (VAS)	Frailty was significantly associated with confusion, malnutrition, risk of sarcopenia, impaired muscle performance, complaints in mobility, in self-care, and in performing usual activities of daily life
De las Penas et al. (2022a), Spain [65] Multicenter cohort study	From 2000 patients randomly selected, 1593 (80.9%) were assessed at T1 and T2 months after hospital discharge	Individuals with a diagnosis of SARS-CoV-2 by RT-PCR technique and radiological findings hospitalized during the first wave of the pandemic	No clear definition of timeframe for evaluating post-COVID syndrome	The Hospital Anxiety and Depression Scale (HADS) The Pittsburgh Sleep Quality Index (PSQI)	Although the prevalence of post-COVID anxiety and depressive symptoms was considerable, a potential recovery over the following months was observed, explaining the downward prevalence trend
De las Penas et al. (2022b), Spain [66] Multicenter cohort study	From 2000 patients randomly selected, a total of 1969 participants (Mean age: 61, SD: 16 years, 46.4% women) were finally included	Individuals with a diagnosis of SARS-CoV-2 by RT-PCR technique and radiological findings hospitalized during the first wave of the pandemic	No clear definition of timeframe for evaluating post-COVID syndrome	The Hospital Anxiety and Depression Scale (HADS) The Pittsburgh Sleep Quality Index (PSQI)	The number of post-COVID symptoms was 2.25 for females and 1.5 for males. After adjusting by all variables, female gender was associated with 3 post-COVID symptoms, the presence of post-COVID fatigue, dyspnea, pain, hair loss, ocular problems, depressive levels, and worse sleep quality
Farooqui et al. (2022), USA [56] Retrospective study	N = 30 individuals with documented COVID-19 illness	Adult patients referred and assessed for psychiatric complications at a university hospital-based post-COVID-19 Recovery Program	Lack of definition of post-COVID syndrome	Physical Health Questionnaire-9 (PHQ-9) Generalized Anxiety Disorder-7 (GAD-7) Columbia Suicide Severity Rating Scale (C-SSRS) Fatigue Severity Scale (FSS) Montreal Cognitive Assessment (MOCA)	A total of 68% of the patient population had a combination of depression and/or anxiety in addition to reported complaints of fatigue and cognitive problems. Out of these, 14 (47%) met the criteria for a primary depressive disorder, followed by 17% (n = 5) who met the criteria for a primary anxiety disorder and 7% (n = 2) who met the clinical criteria for both a depressive disorder and an anxiety disorder
Garout et al. (2022), Saudi Arabia [67] Online survey	N = 744 participants who recovered from COVID-19 disease	Participants declared that they have been diagnosed with COVID-19 by confirmed (SARS-CoV-2) polymerase chain reaction (PCR) Participants who have had COVID-19 at least 2 months before the questionnaire	Post-COVID syndrome defined as having been infected by COVID-19 at least 2 months before	COVID-19 Yorkshire Rehabilitation Screening (C19-YRS)	Out of 744 participants, in 21.4% (N = 189) experienced continual symptoms including anxiety in 13.2% (N = 98) and depression in 9.5% (N = 70)
Gasnier et al. (2022), France [68] Cross-sectional study	N = 170 patients	Age ≥18 years old Hospitalized for >24 h primarily related to COVID-19, with a SARS-CoV-2 infection admitted in intensive care unit during acute phase and/or with at least one long COVID complaint (screened by telephone consultation 4 months after acute COVID-19)	Complaints had to have appeared or worsened since acute COVID-19 infection, and to persist since hospital discharge	Insomnia Severity Index (ISI) Hospital Anxiety and Depression Scale-Anxiety subscale (HAD-A) Beck Depression Inventory-13 items (BDI) PTSD CheckList for Diagnostic and Statistical Manual of Mental Disorders, Fifth Edition (DSM-5) (PCL-5)	Fatigue (44.1%), respiratory complaints (43.5%), cognitive complaints (23.7%), and paraesthesia (20.9%) were the most common long COVID complaints. The number of long COVID complaints was significantly associated with insomnia, anxiety, depression, and post-traumatic stress symptoms. The number of long COVID complaints was greater in patients with a psychiatric disorder, in those with a new-onset psychiatric disorder and in those with a significant suicide risk compared with patients without any past or current psychiatric disorder
Iosifescu et al. (2022), USA [57] Retrospective study	N = 18,811 COVID-19 patients N= 5772 flu patients	COVID-19 patients with neuro-Post Acute Syndrome COVID (PASC) Symptoms COVID-19 patients without neuro-PASC symptoms Flu patients with neurological and neuropsychiatric symptoms	Persistence of symptoms: at least 2 weeks past the date of COVID-19 or flu diagnosis	Clinical records/clinical assessment	Common neuro-PASC symptoms were anxiety (30%), depression (27%), dizziness (22%), altered mental status (17%), chronic headaches (17%), and nausea (11%). The average time to neuro-PASC onset was 138 days
Jozuka et al. (2021), Japan [59] Case report	55-year-old female with COVID-19 accompanied by mild respiratory symptoms showed delusion, psychomotor excitement, and poor communication ability during quarantine outside the hospital.	Not applicable	Lack of specific definition	Clinical records	Case was severe and long-lasting. Neuropsychiatric symptoms after mild respiratory symptoms caused by COVID-19. Numerous residual neuropsychiatric symptoms, such as insomnia, fatigue, loss of concentration, and unsteadiness while walking, which have been reported as neuropsychiatric sequelae of COVID-19. These symptoms were associated also with slow EEG waves, postural tachycardia, and disturbed frontal lobe function
Magdy et al. (2022), Egypt [69] Case–control study	N = 408 patients Group 1: N = 204 COVID-19 survivors with a confirmed history of pre- COVID episodic migraine (migraine headache sufferers) Group 2: N = 204 COVID-19 survivors with no history of any primary headache disorders preceding COVID-19 infection (control)	Older than 18 years Confirmed history of COVID-19 diagnosis by reverse transcription-polymerase chain reaction (RT-PCR) by nasal and oropharynx swabs	After 3 months of severe acute respiratory syndrome coronavirus-2 (SARS-CoV-2) infection	A detailed general, neurological and otolaryngological examination was done for all patients who attended the face-to-face interview. The diagnostic and Statistical Manual of Mental Disorders (DSM-5) was applied for diagnosing insomnia, depression, and anxiety disorders The Montreal Cognitive Assessment (MoCA) The post-COVID-19 Functional Status scale	The reported significant post-COVID-19 neuropsychiatric symptoms in migraine patients compared to controls were fatigue, anosmia/hyposmia, cacosmia, depression, anxiety, insomnia, and headache. There was no statistically significant difference between migraine patients and controls regarding the post-COVID-19 functional status score
Matsumoto et al. (2022), Japan and Sweden [70] International and collaborative cross-sectional study (online).	N = 763 total participants N = 135 infected with COVID-19. N = 628 never been infected with COVID-19	At least 18 years old Data collection carried out through Asmark companies in Japan and Prolific in Sweden online research platforms	Lack of definition of post-COVID syndrome	Ad hoc questionnaire for collecting data on COVID-19 The Fear of COVID-19 Scale (FCV-19S) Patient Health Questionnaire-9 (PHQ-9) General Anxiety Disorder-7 -item (GAD-7) Impact of Event Scale-Revised (IES-R)	For clinically significant syndromes of COVID-19-related anxiety, depression, general anxiety, and PTSD, the proportion of the participants, who exceeded the cut-off on each clinical symptom rating scale, were significantly high in the group that had developed COVID-19 with post-COVID conditions
Ohira et al., (Japan), 2022 [58] Descriptive case series study	N = 90 long COVID patients (39 male, 51 female)	Electronic medical records and clinical summaries of patients who visited the clinic and reported symptoms after recovering from the acute phase of COVID-19 All patients were over 15 years old at the time of their visit At least 2 months had elapsed since the diagnosis of COVID-19 or the end of hospitalization	At least 2 months had elapsed since the diagnosis of COVID-19 or the end of hospitalization	All patients were examined by physicians who were each certified as a Fellow of the Japanese Society of Internal Medicine, and board-certified neurologists of the Japanese Society of Neurology MRI scans were performed using a 3-Tesla MR scanner Olfactory acuity tests used the T&T olfactometer threshold test	The most common chief complaint was disturbance of smell and/or taste (38.9%), followed by memory disturbance (24.4%), fatigue (31.1%), headache (18.9%), hair loss (16.7%), and sleeping problems, including insomnia (13.3%)
Rivera-Izquierdo et al. (2022), Spain [71] Case-control study	N = 906 adult patients. N = 453 patients hospitalized due to COVID-19. N = 453 hospitalized due to other causes.	Randomly selected sample from all hospitalized patients, with laboratory-confirmed SARS-CoV-2 infection through PCR-positive samples		Modified version of the open-access Case Report Form of the Clinical Characterization Protocol for Severe Emerging Infections of the International Severe Acute Respiratory and Emerging Infection Consortium (ISARIC) Medical records	Most frequently occurring symptoms in the COVID-19 cohort were persistent pharyngeal symptoms, confusion or memory loss, thrombotic events, and anxiety. Patients hospitalized due to COVID-19 showed a higher prevalence of respiratory, neurological, and anxiety symptoms after adjusting for sex, age, ICU admission, and baseline comorbidities
Stallmach et al. (2022), Germany [72] Prospective cohort study	N = 355 patients	Symptomatic post-COVID patients who visited out-patient clinics for post COVID-19 care	No clear definition of post-COVID syndrome	Fatigue Assessment Scale, FAS Brief Fatigue Inventory, BFI Depression module of the Patient Health Questionnaire, PHQ-9 Montreal Cognitive Assessment (MoCA) screening) Structured examination consisting of the evaluation of current and initial symptoms, treatment of the SARS-CoV-2 infection, body examination, and amnestic information	Fatigue or signs of depression were reported in 320 patients (90.1% of all patients). Chronic fatigue was found in 93.2% of patients. Depression was reported in 81.3% of patients. Cognitive dysfunction was found in 23.5% of patients
Strahm et al. (2022), Switzerland [73] Prospective observational study	N = 3346 participants.	Hospital employees from 23 healthcare institutions located in northern and eastern Switzerland	“Long post-COVID” = 13 to 24 weeks “Persistent post-COVID” = more than 24 weeks	Rivermead Post Concussion Questionnaire (RMEAD) score 9-item Fatigue Severity Scale (FSS) 8-item Patient Health Questionnaire (PHQ) 7-item General Anxiety Disorder (GAD) score	Symptoms included exhaustion/burnout in 33% of patients, nasopharyngeal swap (NPS)-positive vs. 25% in only seropositive and weakness/tiredness (34% and 25%, respectively). Clinically relevant fatigue was found in 10.6% of the sample
Voruz et al. (2022), Spain [74] Cross-sectional study	N = 102 patients. N = 26 anosognosics patients. N = 76 non-anosognosics patients.	Participants were recruited either via admission lists provided by their treating doctors or from the COVID-COG cohort.	Not a defined time frame for considering a post--COVID condition.	Beck Depression Inventory-Second Edition State–Trait Anxiety Inventory Apathy Motivation Index Posttraumatic Stress Disorder Checklist for DSM-5 Goldberg Mania Inventory Dissociative Experience Scale Perceived Stress Scale	Patients were first divided into two groups according to the to the presence or absence of anosognosia for memory deficits Only 15.6% of patients who presented a mild disease displayed anosognosia for memory dysfunction, compared with 32.4% of patients with a moderate presentation and 34.8% of patients with severe disease.

## Data Availability

Not applicable.
